# Are there gender differences in the geography of alcohol-related mortality in Scotland? An ecological study

**DOI:** 10.1186/1471-2458-9-58

**Published:** 2009-02-16

**Authors:** Carol Emslie, Richard Mitchell

**Affiliations:** 1 MRC Social and Public Health Sciences Unit, Glasgow, UK; 2 Dept of Public Health and Health Policy, Medical Faculty, Glasgow University, Glasgow, UK

## Abstract

**Background:**

There is growing concern about alcohol-related harm, particularly within Scotland which has some of the highest rates of alcohol-related death in western Europe. There are large gender differences in alcohol-related mortality rates in Scotland and in other countries, but the reasons for these differences are not clearly understood. In this paper, we aimed to address calls in the literature for further research on gender differences in the causes, contexts and consequences of alcohol-related harm. Our primary research question was whether the kind of social environment which tends to produce higher or lower rates of alcohol-related mortality is the same for both men and women across Scotland.

**Methods:**

Cross-sectional, ecological design. A comparison was made between spatial variation in men's and women's age-standardised alcohol-related mortality rates in Scotland using maps, Moran's Index, linear regression and spatial analyses of residuals. Directly standardised mortality rates were derived from individual level records of death registration, 2000–2005 (n = 8685).

**Results:**

As expected, men's alcohol-related mortality rate substantially exceeded women's and there was substantial spatial variation in these rates for both men and women within Scotland. However, there was little spatial variation in the relationship between men's and women's alcohol-mortality rates (*r*^2 ^= 0.73); areas with relatively high rates of alcohol-related mortality for men tended also to have relatively high rates for women. In a small number of areas (8 out of 144) the relationship between men's and women's alcohol-related mortality rates was significantly different.

**Conclusion:**

In as far as geographic location captures exposure to social and economic environment, our results suggest that the relationship between social and economic environment and alcohol-related harm is very similar for men and women. The existence of a small number of areas in which men's and women's alcohol-related mortality had an different relationship suggests that some places may have unusual drinking cultures. These might prove useful for further investigations into the factors which influence drinking behaviour in men and women.

## Background

There is growing concern about harmful alcohol consumption in Europe [1], in the United Kingdom [2], and within Scotland in particular [3]. Scotland currently has one of the highest rates of mortality due to cirrhosis of the liver in western Europe [4]. Men's alcohol-related death rates in Scotland rose from 16.1 per 100,000 in the early 1990s to 39.1 deaths per 100,000 a decade later; the comparative rise for women was from 8.1 to 15.7 deaths per 100,000 [5]. These figures illustrate the persistence of substantial gender differences in alcohol-related deaths (and in other medical and social alcohol-related problems). In every society where alcohol use has been studied, men drink more than women [6-9]. However, whilst these stark gender differences in alcohol-related mortality – and consumption – are highly apparent, the reasons for such gender differences are less clearly understood. Wilsnack et al [6] argue that:-

"The lack of adequate explanations for gender differences in drinking behavior reflects the paucity of knowledge about these differences. Despite a growing reservoir of data on drinking practices around the world, few studies go beyond analyses showing that men use and abuse alcohol more than women do. Remarkably little is known about how gender differences in alcohol use and abuse vary or form patterns, across cultures and over time. Even less is known about how women and men may differ in the causes, contexts, and consequences of their drinking behavior." (p253)

In this paper, we address this call for further research on gender differences in the "causes, contexts and consequences of drinking" by using a study design which exploits the ways in which causes and contexts of drinking may vary in geographical space. The close relationship between socio-economic environment and geographical location has long been used as a means to examine how different kinds of life circumstances and environments may be implicated in health [10-13]. As Davidson and colleagues suggest [14], maps of spatial differences in health "are not simply maps of where the sick and the well are to be found, they are maps of the processes and relationships which produce and reproduce 'health"' (p168).

We used a spatial approach to examine gender differences in alcohol-related mortality. The method explored whether the kinds of social environment which are associated with alcohol-related mortality are the same for men and women. It is important to be clear that our main focus was on geographical variation in the relationships *between *men and women's alcohol-related mortality rate, not on the factors which might raise or lower the rate. Our approach did *not *explore why alcohol-related mortality is higher in some areas than others, instead it explored whether the relationship between men and women's alcohol-related mortality rate is the same in different areas of Scotland. To our knowledge, no previous study has adopted this approach in the study of gender differences in alcohol-related health.

There is a general lack of evidence about whether and how the relationship between area and health differs for men and women [15,16] and the little evidence which exists on gender and health outcomes is inconsistent. For example, researchers have found broadly similar patterns among men and women in the relationship between Primary Care Trusts and obesity in England [17] and in the relationship between wards and patterns of suicide in England [18]. In contrast, others have found gender differences in the relationship between postcode sector and self-assessed health in the UK ()[16], in the relationship between living in remote rural areas and suicide mortality in Scotland [19] and Wales [18] and in the relationship between living in rural areas and mortality due to alcohol poisoning in Poland [9]. There is also some evidence that the relationship between perceived neighbourhood problems and health outcomes differs for men and women [16,20,21].

In this study, we expected women's alcohol-related mortality rate to be generally lower than men's, given the existing evidence [5]. We also expected the alcohol-related mortality rate to vary with social and economic context: in other words, some areas would have a higher rate of alcohol-related mortality than others because life circumstances in these areas elevate consumption. However, given the lack of previous work on this topic, our research on the relationship between gender and the spatial patterning of alcohol-related deaths is exploratory. It could be argued that environments which produce hazardous drinking men, and hence a higher rate of alcohol-related mortality in men, will be the same as those which produce a higher rate of alcohol-related mortality in women. Alternatively, local area differences in gendered drinking cultures, or in the gender norms which influence whether men and women seek help for problem drinking, might lead to different relationships between the alcohol-related mortality rate of men and women in different areas. Therefore, our research questions were:-

• How does the alcohol-related mortality rate among men and among women vary geographically in Scotland?

• Does the relationship between men's and women's alcohol-related mortality rate vary across Scotland?

## Methods

### Areal units

**Figure 1:**
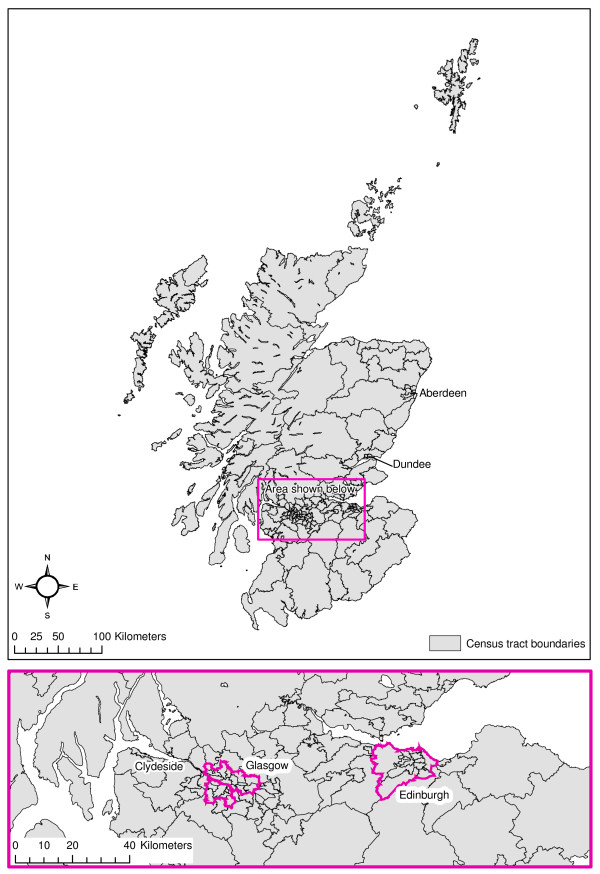
**Census tract boundaries in Scotland**.

The selection of areal units has considerable implications for spatial analyses [22]. For this study, we needed areal units which were physically small enough to reflect spatial variation in the mortality rate across Scotland but had large enough populations to provide robust estimates of alcohol-related mortality for men and women separately. In particular, we used directly standardised mortality rates in our analyses because we wanted to compare 'absolute' mortality rates for men and women, rather than the relative standardised mortality ratio produced by indirect standardisation. However, because directly standardised rates are more sensitive to small populations, areal unit population size was particularly important. We therefore selected census tracts specifically designed for the investigation of geographical inequalities in mortality in the UK [23]. These divide Scotland into 144 units with an average population of c35,000 people and have the advantage of fragmenting Scotland's larger urban areas but also retaining reasonable spatial division of the extensive and sparsely populated rural Highlands. Figure 1 shows census tracts in Scotland with some key areas labelled. The more densely populated area which lies on an axis between Glasgow in the west and Edinburgh in the east is enlarged.

### Mortality Rates

We derived population denominators for gender specific 5-year age groups for census tracts from the General Register Office for Scotland (GRO(S))population estimates for 2003 [24]. Mortality data were individual-level geocoded mortality records made available by GRO(S). These were matched to census tract and then grouped to match the age-sex structure of the denominator data. Our definition of an 'alcohol-related death' followed the standard set by the Office of National Statistics [5]. The International Classification of Diseases (10) codes used were: F10, G31.2, G62.1, G62.1, I42.6, K29.2, K70, K73, K74 (excluding K74.3–K74.5), K86.0, X45, X65, Y15. We aggregated deaths registered in 2000–2005 and adjusted for this aggregation in the rate calculations (effectively taking the average number of deaths as the numerator). We then produced directly standardised rates for alcohol-related mortality (standardised to the European population).

### Analyses

We first mapped the rates using ArcMap 9 and visually examined the patterns. We then calculated the Morans I statistic [25] for both men and women. This is a formal test for spatial autocorrelation of high or low mortality rates and indicates the extent to which the distribution of either may be random or spatially concentrated. Next, we examined the relationship between the age-standardised alcohol-related mortality rate for women and men using a scatter plot, confirming its suitability for linear regression. We built a linear regression model in which women's rate was predicted by men's and derived predicted values and residuals from this model. These residuals represented a measure of the deviation from a 'usual' relationship between men and women's alcohol-related mortality rate and they were explored in two ways. We first mapped all of them and calculated a Morans I statistic. We then formally identified those areas with statistically significant outlier residuals (i.e. in which the relationship between men's and women's age-standardised alcohol-related mortality rate was significantly different to the average).

We subsequently examined the relationship between both mortality rates and the model residuals, and two measures of socio-economic deprivation, in order to determine whether those areas in which the relationship between men's and women's alcohol-related mortality was unusual were also characterised by particular levels of deprivation. Our first area measure of socio-economic deprivation was the Breadline Britain 2001 index which estimates the proportion of a population living below the poverty line [26]. However, we also wanted to use gender-specific measures of socio-economic deprivation to determine whether those areas in which the relationship between men's and women's alcohol-related mortality was unusual were characterised by particular relationships between men's and women's socio-economic status. To our knowledge, no well validated and commonly used gender-specific measure of socio-economic deprivation is available. We thus produced a simple one ourselves by using the proportion of working age men or women classified by the 2001 UK decennial Census as being in NS-SEC (National Statistics Socioeconomic Classification) group 7 (routine occupations) or group 8 (never worked or long-term unemployed) in each tract. NS-SEC is a widely used occupation-based socio-economic indicator introduced in the UK in 2001 in order to reflect changes in the labour market and the increasing number of women in paid employment. (Tracts had very similar proportion of men and women in NS-SEC group 7 or 8 (*r*^2 ^= 0.92, p < 0.0001)). We explored these measures separately and also calculated the ratio between them as a crude proxy for 'inequality' between men and women's socio-economic status. (As an alternative measure, we used levels of educational achievement by men and women in each tract. Results were the same as for NS-SEC; data not shown). All statistical analyses were carried out in Stata 10. Ethical approval was not required for this secondary analysis of anonymised, routinely recorded data.

## Results

### Overview

There were 8685 alcohol-related deaths in Scotland between 2000 and 2005 (an average of 1448 per year). These deaths accounted for about 3% of all-cause mortality in Scotland. As expected, there were substantial gender differences; there were 5995 (999 a year: rounded) alcohol-related deaths among men and 2690 (448) among women, accounting for 4% of all male deaths and 1% of all female deaths. The all-age directly standardised rate of alcohol-related mortality was 38.0 per 100,000 population for men and 15.5 per 100,000 for women.

### Geographical variation in alcohol-related mortality rates

**Figure 2:**
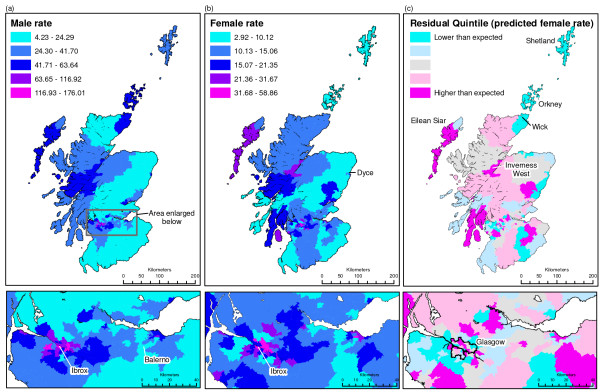
**Male and female age-standardised alcohol-related mortality rate at census tract level (2000–2005) and residuals from regression model predicting women's rate from men's rate**.

Figures 2a and 2b illustrate the wide geographical variation in mortality rate among men and among women respectively. They also show that many areas with the highest alcohol-related mortality rates were located within Greater Glasgow. For example, men's age-standardised rate of alcohol-related mortality ranged from 4.2 per 100,000 in Balerno (a suburb of Edinburgh) to 176 per 100,000 in Glasgow Ibrox, while women's ranged from 2.9 per 100,000 in Dyce (a suburb of Aberdeen) to 58.9 per 100,000 in Glasgow Ibrox. (These areas are labelled in figure 2). For both men and women, the Morans I statistic confirmed significant spatial clustering of values. A Moran's Index value near +1.0 indicates total clustering and an index value near -1.0 indicates dispersion. The value for men was 0.28 (p < 0.01) and for women it was 0.24 (p < 0.01). Additional file 1 lists the alcohol-related mortality rate for men and women for all 144 areas in the study.

### Geographical variation in the relationship between men and women's mortality rate

The regression model predicting women's alcohol mortality rate from men's had an *r*^2 ^of 0.73 (p < 0.001). Figure 2c maps all the residuals derived from this model. The dark pink areas indicate the top quintile of residuals (areas where the rate of alcohol-related mortality was higher than expected for women relative to men), while the turquoise areas indicate the bottom quintile (areas where women's rate was lower than expected relative to men). There was a weak suggestion that women's alcohol-related mortality rate was lower than expected relative to men in Eastern coastal areas (including Orkney and Shetland). However, there was no clear geographical pattern and certainly no clear rural/urban split. For example, women had a much higher mortality rate than expected relative to men in some areas of Glasgow (the city boundary is shown on the map), but a much lower mortality rate than expected in other contiguous urban areas. Similarly, women had a higher rate than expected relative to men in some rural areas, such as Inverness West and Eilean Siar rural, but a lower rate than expected in Orkney, Wick and Shetland. (These areas are labelled on figure 2c). The Moran's I Index for the residuals was 0.01 (p > 0.10). This value, close to 0, indicated a relatively random distribution of values.

**Figure 3:**
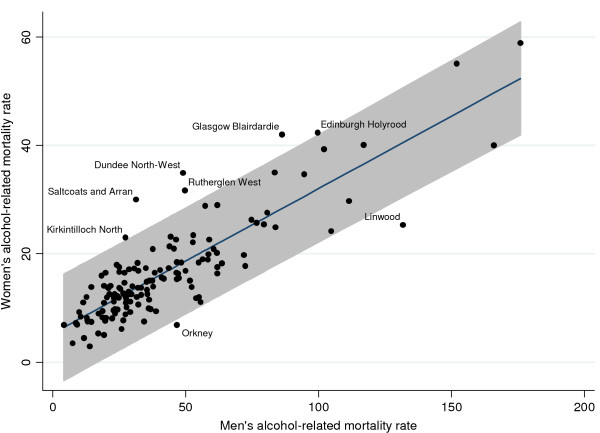
**Scatter plot of men's alcohol-related mortality rate against women's alcohol-related mortality rate, with a regression line fitted, confidence interval for the estimates and significant residuals labelled**.

Figure 3 shows a scatter plot illustrating the relationship between men and women's alcohol-related mortality rates and the regression line from which the residuals were derived. The plot confirms that there is a strong relationship between men's and women's rates but also reveals that there is some variation in this relationship. This variation can be illustrated by comparing two areas in which the rates were similar for men, but very different for women. In Dundee North-West, there were 49 deaths per 100,000 for men compared to 35 per 100,000 for women, while in Orkney there were 47 deaths per 100,000 for men compared to only 7 per 100,000 for women.

The 95% confidence interval around the regression estimates in figure 3 constitutes a formal test for significant outliers in the relationship. Women had significantly 'unusual' alcohol-related mortality rates, relative to men, in only 8 areas (out of a total of 144). In six areas, the alcohol-related mortality rate was particularly high for women, given men's mortality rate in the area, and in two areas the rate for women was much lower than would be expected. There was no clear geographical pattern or clustering to these 'exceptional' areas and they constituted a mixture of area types from very urban through suburban to very rural. The ratio for female: male deaths in these areas varied from 1:1.0 in Saltcoats and Arran (31 and 30 per 100,000 for men and women respectively) to 1: 6.8 in Orkney (47 and 7 per 100,000 deaths for men and women respectively).

We did not find robust evidence of an association between model residuals and measures of deprivation. There was no significant correlation between residuals and the proportion of men or women in NS-SEC groups 7 and 8 (*r*^2 ^= 0.16, p = 0.06 and *r*^2 ^= 0.15, p = 0.07 respectively) or the ratio of these proportions (*r*^2 ^= -0.01, p = 0.9). There was a weak but significant association between the residuals and the Breadline Britain index (*r*^2 ^= 0.21, p < 0.01) but a scatter plot of residuals against the index suggested that this was likely to be a symptom of poorer model performance at higher levels of deprivation. A re-examination of the correlation for those tracts in which the residual was statistically significant confirmed that there was no meaningful association with the Breadline Britain index (*r*^2 ^= 0.00).

## Discussion

In this paper, we aimed to fill a gap in the literature by exploring the relationship between gender and alcohol-related mortality in Scotland. We found substantial spatial variation in the alcohol-related mortality rates of both men and women; areas with higher and lower alcohol-related mortality rates tended to be spatially clustered. Our results confirm previous findings of very high alcohol-related mortality rates in Greater Glasgow [3,5] and fit with reports of elevated levels of 'binge' drinking among men and women in Glasgow City compared with the rest of Scotland, after controlling for socio-economic factors [27].

We also found that women's alcohol-related mortality rate at an area level was strongly predicted by men's. Indeed, in 94% of the areas examined, the relationship between alcohol-related mortality rates for men and women lay within variation which could be reasonably expected by chance. This suggests that, in most places, similar processes and factors are important in determining the risk of mortality from alcohol for both men and women. However, in a small number of places, women's alcohol-related mortality rates were substantially different to those we would expect given men's rates. There was no clear evidence that these areas were characterised by particularly high or low levels of alcohol-related mortality among men or of socio-economic deprivation, and they were not spatially clustered.

Our results have interesting resonances with the findings of Dorling & Gunnell [28]. They constructed a model which very successfully predicted the number of deaths due to suicide across the UK with the exception of a small number of areas with 'geographically unique stories to tell' including remote rural areas of Scotland and some of the poorest parts of Glasgow (p443). This leads us to speculate about the cultural and geographical factors which might result in the development of unusual drinking cultures in the few areas in which women's alcohol-related mortality rates were not predicted by men's. It could be that constructions of 'masculinity' which emphasise physical and mental toughness, endurance and risk-taking – including excessive drinking – are more pervasive and powerful in areas in which historically men have worked in heavy industry, fishing or farming [29]. Data from the 1981 census showed, for example, that Linwood and Orkney (two of the areas in which we found an unusual relationship between men and women's alcohol-related mortality rates) were, for men, dominated by employment in manufacturing and agriculture respectively. In these areas, it is plausible that excessive drinking was encouraged or tolerated among men but viewed much more negatively among women (see, for example, [30,31]) resulting in lower than expected alcohol-related mortality among women, relative to men. It has also been suggested that women's drinking rates are higher in certain types of employment because of different occupational cultures and job stresses [30]. For example, Hartford et al [31] found higher levels of alcohol dependence among female machine operators, labourers, and service workers than women in other occupations. In our study, relatively high proportions of women worked in service industries in 3 of the 6 areas where alcohol-related mortality rates were particularly high for women, given men's rate in the area (Glasgow Blairdardie, Kirkintilloch North and Edinburgh Holyrood). Whilst the census permits such observations about characteristics of local labour markets, there were too few 'unusual' areas to permit robust quantitative investigation and the census is limited in the extent to which it can really capture local cultures and gendered experiences. Qualitative work would be necessary to explore relationships between place, gendered identities and alcohol in a more detailed way in these few 'exceptional' places.

We do not know of any other analysis which has explored the relationship between the spatial patterning of men and women's alcohol-related mortality rates at such a fine scale. Our use of census tracts allowed us to make detailed comparisons within regions which are often treated as single areal units. In particular, the Highlands and Islands of Scotland are often treated as a single region; our analyses showed interesting differences within this region in both alcohol-related mortality rates and the relationships between these rates for men and women. However, like most areal units, census tracts have little substantive meaning in terms of delineating community or cultural boundaries. We have no way of knowing the extent to which these boundaries may delineate 'drinking cultures' for example. Tracts are also still relatively large units which, whilst offering us the advantage of statistical robustness, may have masked important variation at even finer spatial scales. All ecological analyses are also subject to the modifiable areal unit problem [22]. There are other limitations to our study; we only included deaths for which geographical registration was complete (although this only excluded 0.26% of deaths) and it is also possible that geographical variation in death classification might influence our results. In addition, our decision to use age-standardised death-rates means that we have not attempted to unpack any relationships which may exist between age, gender and the spatial patterning of alcohol-related mortality; this should be the focus of future work.

It could be argued that our outcome (alcohol-related death) is more robust than many other measures of problematic alcohol consumption. Time-series analyses suggest that alcohol-related mortality correlates highly with mean alcohol consumption, with very little time lag [9]. In addition, areas which had high rates of alcohol-related mortality in our analysis also tended to have high alcohol-related hospital discharge rates [32]. Recent research suggests that surveys may underestimate alcohol consumption by as much as 50% [33] so using mortality as an outcome avoids many biases inherent in self-reported measures. However, it is possible that other confounding factors such as obesity may influence the relationship between drinking and alcohol-related mortality [4].

## Conclusion

It is well known that gender differences in alcohol use and alcohol-related harm occur consistently across cultures and over time, and that place is a powerful predictor of health and death. However, relatively little work has attempted to simultaneously explore the influence of both gender and geographical space. In this paper, we found very few places in which women's alcohol-related mortality rate was substantially different to what we might expect, given men's rate. Further research on the few 'exceptional' places that our analysis identified might be a valuable way to explore unusual drinking cultures and so contribute to understandings about gender and alcohol use. Our main finding was that women's alcohol-related mortality rate was strongly predicted by men's rate, suggesting that similar factors within social environments are important in influencing the risk of alcohol deaths among both men and women. Areas which have a high rate of alcohol-related deaths for men are very likely to also have a high rate of alcohol-related deaths for women. Thus, while area of residence appears to be associated with the amount that men and women drink in Scotland, it seldom influences the 'gender gap' in drinking; in other words, the factors associated with gender differences in drinking are so powerful that they seem to transcend socio-economic environment.

## Competing interests

The authors declare that they have no competing interests.

## Authors' contributions

CE and RM conceived the study. RM analysed the data and CE wrote the first draft. Both authors helped to refine the analysis, contributed to the final version of the paper and approved the final manuscript. The authors have made an equal contribution to the paper.

## Pre-publication history

The pre-publication history for this paper can be accessed here:



## Supplementary Material

Additional file 1 **Appendix.** Age-standardised alcohol-related mortality rates in Scotland per 100,000 population, by gender (2000–2005) Click here for file
