# Supplementary material for: Are there gender differences in the geography of alcohol-related mortality in Scotland? An ecological study
Source: BMC Public Health. 2009 Feb 16;9:58. doi: 10.1186/1471-2458-9-58 (PMC2652460; doi:10.1186/1471-2458-9-58)
Supplement: Additional file 1 — Appendix. Age-standardised alcohol-related mortality rates in Scotland per 100,000 population, by gender (2000–2005) [file 1471-2458-9-58-S1.doc]

**Additional file 1: Age-standardised alcohol-related mortality rates in Scotland per 100,000 population, by gender (2000-2005)**

| ***Sorted by rates for men*** | | |  | ***Sorted by rates for women*** | | |
| --- | --- | --- | --- | --- | --- | --- |
| **Area Name** | **Men** | **Women** |  | **Area Name** | **Men** | **Women** |
| Glasgow Ibrox | 176.0 | 58.9 |  | Glasgow Ibrox | 176.0 | 58.9 |
| Glasgow Calton | 166.1 | 40.0 |  | Glasgow Cowlairs | 152.1 | 55.0 |
| Glasgow Cowlairs | 152.1 | 55.0 |  | Edinburgh Holyrood | 99.7 | 42.4 |
| Linwood | 131.8 | 25.3 |  | Glasgow Blairdardie | 86.3 | 42.0 |
| Glasgow University | 116.9 | 40.1 |  | Glasgow University | 116.9 | 40.1 |
| Glasgow Easterhouse | 111.5 | 29.7 |  | Glasgow Calton | 166.1 | 40.0 |
| Greenock North | 104.8 | 24.1 |  | Glasgow Milton | 102.1 | 39.3 |
| Glasgow Milton | 102.1 | 39.3 |  | Glasgow Maryhill | 83.5 | 35.0 |
| Edinburgh Holyrood | 99.7 | 42.4 |  | Dundee North-West | 49.0 | 34.9 |
| Glasgow Parkhead | 94.6 | 34.7 |  | Glasgow Parkhead | 94.6 | 34.7 |
| Glasgow Blairdardie | 86.3 | 42.0 |  | Rutherglen West | 49.7 | 31.7 |
| Johnstone | 83.8 | 24.9 |  | Saltcoats and Arran | 31.3 | 30.0 |
| Glasgow Maryhill | 83.5 | 35.0 |  | Glasgow Easterhouse | 111.5 | 29.7 |
| Dundee South-East | 80.7 | 27.6 |  | Inverness West | 61.9 | 28.9 |
| Glasgow Nitshill | 79.4 | 25.4 |  | Edinburgh East | 57.4 | 28.9 |
| Glasgow Robroyston | 76.7 | 25.7 |  | Dundee South-East | 80.7 | 27.6 |
| Glasgow Cardonald | 74.8 | 26.2 |  | Glasgow Cardonald | 74.8 | 26.2 |
| Glasgow Partick | 72.5 | 17.8 |  | Glasgow Robroyston | 76.7 | 25.7 |
| Clydebank | 71.9 | 19.7 |  | Glasgow Nitshill | 79.4 | 25.4 |
| Old Aberdeen | 63.6 | 18.2 |  | Linwood | 131.8 | 25.3 |
| Stirling Urban | 61.9 | 17.5 |  | Johnstone | 83.8 | 24.9 |
| Paisley Blackhall | 61.9 | 16.3 |  | Greenock North | 104.8 | 24.1 |
| Inverness West | 61.9 | 28.9 |  | Falkirk Central | 52.8 | 23.4 |
| Glasgow Castlemilk | 61.8 | 20.2 |  | Eilean Siar Rural | 44.3 | 23.2 |
| Glasgow Knightswood | 60.6 | 20.9 |  | Kirkintilloch North | 27.3 | 23.0 |
| Leith | 58.9 | 22.6 |  | Blantyre | 46.3 | 22.6 |
| Dumbarton Central | 58.5 | 19.9 |  | Leith | 58.9 | 22.6 |
| Ayr Central | 58.4 | 18.9 |  | Wishaw | 52.7 | 22.1 |
| Edinburgh East | 57.4 | 28.9 |  | Hamilton North | 44.0 | 21.4 |
| Motherwell | 56.3 | 19.0 |  | Glasgow Pollokshields | 45.6 | 21.0 |
| Wick | 55.6 | 11.0 |  | Paisley Gallowhill | 37.7 | 20.9 |
| Barrhead | 55.1 | 12.0 |  | Glasgow Knightswood | 60.6 | 20.9 |
| Port Glasgow | 54.9 | 18.4 |  | Glasgow Castlemilk | 61.8 | 20.2 |
| Greenock South | 54.0 | 11.8 |  | Dumbarton Central | 58.5 | 19.9 |
| Falkirk Central | 52.8 | 23.4 |  | Clydebank | 71.9 | 19.7 |
| Wishaw | 52.7 | 22.1 |  | Motherwell | 56.3 | 19.0 |
| Edinburgh North | 52.3 | 13.9 |  | Ayr Central | 58.4 | 18.9 |
| Aberdeen Nigg Bay | 51.6 | 15.0 |  | Mallaig | 46.7 | 18.4 |
| Shotts | 50.0 | 16.8 |  | Port Glasgow | 54.9 | 18.4 |
| Rutherglen West | 49.7 | 31.7 |  | Rutherglen East | 48.4 | 18.4 |
| Dundee North-West | 49.0 | 34.9 |  | Bridge of Allan | 31.9 | 18.3 |
| Rutherglen East | 48.4 | 18.4 |  | Old Aberdeen | 63.6 | 18.2 |
| Perth Central | 47.5 | 15.5 |  | Livingston Central | 24.0 | 18.0 |
| Leven | 47.1 | 16.3 |  | Glasgow Partick | 72.5 | 17.8 |
| Edinburgh Murrayfield | 46.7 | 15.4 |  | Musselburgh | 25.0 | 17.5 |
| Orkney | 46.7 | 6.9 |  | Stirling Urban | 61.9 | 17.5 |
| Mallaig | 46.7 | 18.4 |  | Cumbernauld | 35.1 | 17.4 |
| Coatbridge | 46.5 | 16.4 |  | Belshill | 43.7 | 17.3 |
| Blantyre | 46.3 | 22.6 |  | Midlothian South-East | 30.7 | 17.2 |
| Glasgow Pollokshields | 45.6 | 21.0 |  | Bute | 28.7 | 17.1 |
| Eilean Siar Rural | 44.3 | 23.2 |  | Linlithgow South | 40.4 | 17.0 |
| Hamilton North | 44.0 | 21.4 |  | Sidlaw and Carnoustie | 32.2 | 16.8 |
| Belshill | 43.7 | 17.3 |  | Shotts | 50.0 | 16.8 |
| Kilwinning | 41.7 | 15.4 |  | Kilmarnock Central | 25.0 | 16.6 |
| Dundee North-East | 41.0 | 15.6 |  | Edinburgh Corstorphine | 38.4 | 16.5 |
| Linlithgow South | 40.4 | 17.0 |  | Forfar | 19.8 | 16.5 |
| Elgin | 38.9 | 9.3 |  | Kilmarnock Rural | 27.0 | 16.5 |
| Edinburgh Corstorphine | 38.4 | 16.5 |  | Coatbridge | 46.5 | 16.4 |
| Stornoway | 37.8 | 13.9 |  | Paisley Blackhall | 61.9 | 16.3 |
| Paisley Gallowhill | 37.7 | 20.9 |  | Leven | 47.1 | 16.3 |
| Glasgow Baillieston | 37.5 | 15.0 |  | Denny | 18.4 | 15.9 |
| Dundee South-West | 36.8 | 9.8 |  | Dundee North-East | 41.0 | 15.6 |
| Edinburgh Sighthill | 36.3 | 15.1 |  | Perth Central | 47.5 | 15.5 |
| Chryston | 36.2 | 11.5 |  | Kilwinning | 41.7 | 15.4 |
| Montrose and Arbroath | 36.1 | 9.9 |  | Edinburgh Murrayfield | 46.7 | 15.4 |
| Larkhall | 35.7 | 12.6 |  | Edinburgh Sighthill | 36.3 | 15.1 |
| Largs and Cumbrae | 35.5 | 13.3 |  | Aberdeen Nigg Bay | 51.6 | 15.0 |
| Cumnock & Doon Valley | 35.4 | 14.9 |  | Glasgow Baillieston | 37.5 | 15.0 |
| Cumbernauld | 35.1 | 17.4 |  | Cumnock & Doon Valley | 35.4 | 14.9 |
| East Kilbride North | 34.3 | 7.5 |  | Kirkcaldy North | 26.9 | 14.6 |
| Airdrie | 33.4 | 13.7 |  | Caithness | 19.3 | 14.1 |
| Edinburgh Kaimes | 33.3 | 12.4 |  | Lanark | 21.7 | 14.1 |
| Sidlaw and Carnoustie | 32.2 | 16.8 |  | Irvine | 30.0 | 14.0 |
| Prestonpans | 32.1 | 10.6 |  | Stornoway | 37.8 | 13.9 |
| Livingston Rural | 32.0 | 13.7 |  | Edinburgh North | 52.3 | 13.9 |
| Bridge of Allan | 31.9 | 18.3 |  | Ettrick and Lauderdale | 14.4 | 13.8 |
| Falkirk Laurieston | 31.5 | 12.0 |  | Dumfries Central | 20.7 | 13.8 |
| Saltcoats and Arran | 31.3 | 30.0 |  | Airdrie | 33.4 | 13.7 |
| Midlothian South-East | 30.7 | 17.2 |  | Livingston Rural | 32.0 | 13.7 |
| Irvine | 30.0 | 14.0 |  | Inverness East | 25.1 | 13.7 |
| East Kilbride South | 29.5 | 12.5 |  | Helensburgh | 21.1 | 13.6 |
| Bridge of Weir | 29.1 | 9.2 |  | Dunfermline Central | 25.5 | 13.5 |
| Glasgow Newlands | 29.1 | 11.2 |  | Largs and Cumbrae | 35.5 | 13.3 |
| Bute | 28.7 | 17.1 |  | Dingwall and Skye | 28.5 | 13.0 |
| Dingwall and Skye | 28.5 | 13.0 |  | Midlothian North-West | 26.5 | 12.7 |
| Forres | 28.4 | 12.5 |  | Larkhall | 35.7 | 12.6 |
| Lorn | 27.8 | 10.1 |  | Aberdeen West | 23.3 | 12.6 |
| Linlithgow North | 27.6 | 11.6 |  | Nithsdale | 21.3 | 12.5 |
| Cowdenbeath | 27.5 | 10.9 |  | East Kilbride South | 29.5 | 12.5 |
| Hamilton Cadzow | 27.4 | 12.1 |  | Forres | 28.4 | 12.5 |
| Prestwick and Troon | 27.4 | 8.8 |  | Edinburgh Kaimes | 33.3 | 12.4 |
| Kirkintilloch North | 27.3 | 23.0 |  | Dunfermline North-East | 24.2 | 12.2 |
| Kilmarnock Rural | 27.0 | 16.5 |  | Hamilton Cadzow | 27.4 | 12.1 |
| Kirkcaldy North | 26.9 | 14.6 |  | Falkirk Laurieston | 31.5 | 12.0 |
| Kirkcaldy South | 26.5 | 7.7 |  | Newburgh | 12.7 | 12.0 |
| Midlothian North-West | 26.5 | 12.7 |  | Barrhead | 55.1 | 12.0 |
| Shetland | 25.9 | 6.1 |  | Glenrothes | 20.4 | 11.9 |
| Dunfermline Central | 25.5 | 13.5 |  | Kilsyth | 23.0 | 11.9 |
| Inverness East | 25.1 | 13.7 |  | Dunfermline South-West | 24.8 | 11.8 |
| Kilmarnock Central | 25.0 | 16.6 |  | Greenock South | 54.0 | 11.8 |
| Musselburgh | 25.0 | 17.5 |  | Linlithgow North | 27.6 | 11.6 |
| Dunfermline South-West | 24.8 | 11.8 |  | Chryston | 36.2 | 11.5 |
| Buchan | 24.3 | 9.7 |  | Alloa | 23.0 | 11.2 |
| Dunfermline North-East | 24.2 | 12.2 |  | Glasgow Newlands | 29.1 | 11.2 |
| Livingston Central | 24.0 | 18.0 |  | Wick | 55.6 | 11.0 |
| Edinburgh Morningside | 23.5 | 9.8 |  | Stirling Rural | 11.4 | 11.0 |
| Crieff | 23.5 | 8.9 |  | Cowdenbeath | 27.5 | 10.9 |
| Aberdeen West | 23.3 | 12.6 |  | Haddington | 19.3 | 10.9 |
| Alloa | 23.0 | 11.2 |  | Prestonpans | 32.1 | 10.6 |
| Kilsyth | 23.0 | 11.9 |  | Lorn | 27.8 | 10.1 |
| Grangemouth | 22.9 | 9.6 |  | Montrose and Arbroath | 36.1 | 9.9 |
| Banff | 22.3 | 8.1 |  | Edinburgh Morningside | 23.5 | 9.8 |
| Lanark | 21.7 | 14.1 |  | Dundee South-West | 36.8 | 9.8 |
| Nithsdale | 21.3 | 12.5 |  | Buchan | 24.3 | 9.7 |
| Helensburgh | 21.1 | 13.6 |  | Grangemouth | 22.9 | 9.6 |
| Dumfries Central | 20.7 | 13.8 |  | Aberdeen South-West | 18.6 | 9.4 |
| Glenrothes | 20.4 | 11.9 |  | Elgin | 38.9 | 9.3 |
| Stranraer | 20.1 | 8.2 |  | Bridge of Weir | 29.1 | 9.2 |
| Forfar | 19.8 | 16.5 |  | Coupar Angus | 10.0 | 9.2 |
| Bearsden and Kirkintilloch S | 19.7 | 7.6 |  | Turriff | 17.2 | 9.0 |
| Tweeddale | 19.3 | 5.1 |  | Crieff | 23.5 | 8.9 |
| Caithness | 19.3 | 14.1 |  | Prestwick and Troon | 27.4 | 8.8 |
| Haddington | 19.3 | 10.9 |  | Milngavie | 10.6 | 8.3 |
| St Andrews | 19.2 | 8.2 |  | Carrick | 18.7 | 8.2 |
| Carrick | 18.7 | 8.2 |  | St Andrews | 19.2 | 8.2 |
| Aberdeen South-West | 18.6 | 9.4 |  | Stranraer | 20.1 | 8.2 |
| Denny | 18.4 | 15.9 |  | Berwickshire | 12.9 | 8.1 |
| Turriff | 17.2 | 9.0 |  | Banff | 22.3 | 8.1 |
| Aberdeen Queens Cross | 17.0 | 5.3 |  | Kirkcaldy South | 26.5 | 7.7 |
| Giffnock | 14.5 | 7.4 |  | Queensferry | 13.0 | 7.7 |
| Ettrick and Lauderdale | 14.4 | 13.8 |  | Bearsden and Kirkintilloch S | 19.7 | 7.6 |
| Dyce | 13.8 | 2.9 |  | East Kilbride North | 34.3 | 7.5 |
| Roxburgh | 13.2 | 7.5 |  | Roxburgh | 13.2 | 7.5 |
| Queensferry | 13.0 | 7.7 |  | Giffnock | 14.5 | 7.4 |
| Berwickshire | 12.9 | 8.1 |  | Inverurie | 8.6 | 7.2 |
| Newburgh | 12.7 | 12.0 |  | Banchory | 9.1 | 6.9 |
| Dumfries East | 11.6 | 4.4 |  | Orkney | 46.7 | 6.9 |
| Stirling Rural | 11.4 | 11.0 |  | Balerno | 4.2 | 6.9 |
| Milngavie | 10.6 | 8.3 |  | Shetland | 25.9 | 6.1 |
| Coupar Angus | 10.0 | 9.2 |  | Aberdeen Queens Cross | 17.0 | 5.3 |
| Banchory | 9.1 | 6.9 |  | Tweeddale | 19.3 | 5.1 |
| Inverurie | 8.6 | 7.2 |  | Dumfries East | 11.6 | 4.4 |
| Stonehaven | 7.5 | 3.5 |  | Stonehaven | 7.5 | 3.5 |
| Balerno | 4.2 | 6.9 |  | Dyce | 13.8 | 2.9 |
| ***SCOTLAND*** | ***38.0*** | ***15.5*** |  | ***SCOTLAND*** | ***38.0*** | ***15.5*** |
